# Supplementary material for: NAD+ depletion is central to placental dysfunction in an inflammatory subclass of preeclampsia
Source: Life Sci Alliance. 2024 Oct 10;7(12):e202302505. doi: 10.26508/lsa.202302505 (PMC11467044; doi:10.26508/lsa.202302505)
Supplement: Supplementary file 1 [file LSA-2023-02505_SdataF2.pptx]

## Slide 1
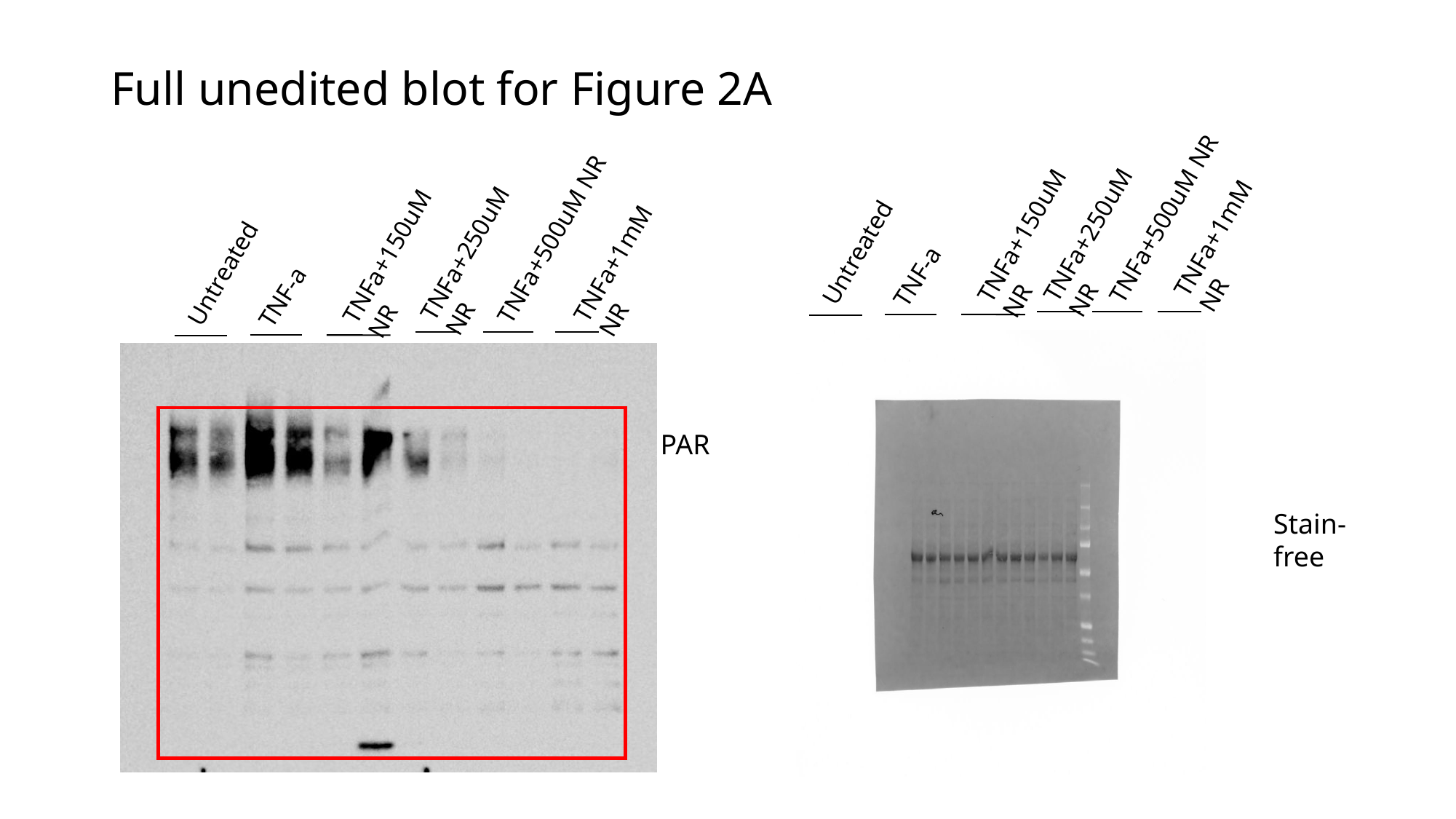

# Full unedited blot for Figure 2A
TNFa+500uM NR
TNFa+150uM NR
TNFa+250uM NR
TNFa+1mM NR
TNF-a
Untreated
TNFa+500uM NR
TNFa+150uM NR
TNFa+250uM NR
TNFa+1mM NR
TNF-a
Untreated
PAR
Stain-free
